# Clinical outcomes in a subpopulation of adults with Morquio A syndrome: results from a long-term extension study of elosulfase alfa

**DOI:** 10.1186/s13023-017-0634-0

**Published:** 2017-05-23

**Authors:** D. Hughes, R. Giugliani, N. Guffon, S. A. Jones, K. E. Mengel, R. Parini, R. Matousek, S. M. Hawley, A. Quartel

**Affiliations:** 1 Royal Free London NHS Foundation Trust and University College London, London, UK; 2 Medical Genetics Service, Hospital de Clínicas de Porto Alegre and Department of Genetics/Universidade Federal do Rio Grande do Sul and Instituto Nacional de Genética Médica Populacional, Porto Alegre, Brazil; 3 Hôpital Femme Mère Enfant, Reference Centre of Inherited Metabolic Diseases, Hospices Civils de Lyon, Lyon, France; 4 Manchester Centre for Genomic Medicine, St Mary’s Hospital, Central Manchester University Hospitals NHS Foundation Trust, University of Manchester, Manchester, UK; 5 Villa Metabolica, Centre for Pediatric and Adolescent Medicine, University of Mainz Medical Center, Mainz, Germany; 6 Fondazione Monza e Brianza per il Bambino e la sua Mamma, Azienda Ospedaliera San Gerardo, Monza, Italy; 7 BioMarin Pharmaceutical Inc, Novato, CA USA

**Keywords:** Morquio syndrome A, Adults, Adulthood, Advanced disease, Long-term, Enzyme replacement therapy, Elosulfase alfa, Mucopolysaccharidosis IVA

## Abstract

**Background:**

This post hoc subanalysis examined outcomes in adult patients with Morquio A (mucopolysaccharidosis IVA) who received enzyme replacement therapy (ERT) with elosulfase alfa over a 120-weeks period. Patients ≥18 years of age evaluated in an open-label, long-term extension study of elosulfase alfa (modified per protocol [MPP], *n* = 32; intent-to-treat [ITT], *n* = 37; MOR-005; NCT01415427) were compared with the ≥18-year-old untreated population with 2-years follow-up from a Morquio A natural history study (*n* = 10; MorCAP; NCT00787995). The MOR-005 MPP population excluded patients who underwent orthopedic surgical procedures or were noncompliant with study protocol (defined as missing ≥20% of ERT infusions). No MorCAP patients underwent orthopedic surgical procedures during the relevant time period. Endurance was assessed by the 6-min walk test (6MWT) and 3-min stair climb test (3MSCT). Activities of daily living (ADLs) were assessed by the MPS Health Assessment Questionnaire (MPS HAQ).

**Results:**

Least squares (LS) mean (SE) 6MWT distances increased by 34.9 (11.7) m (MPP) and 30.5 (10.8) m (ITT) by week 120; LS mean (SE) change in 3MSCT at week 120 was 6.7 (1.8) stairs/min (MPP) and 5.9 (1.7) stairs/min (ITT). MorCAP patients showed no improvement in 6MWT distance or 3MSCT over a similar period of time. Pulmonary function measures remained unchanged in both MOR-005 and MorCAP adults. All MPS HAQ domain scores improved in MOR-005 adults, whereas MorCAP adults had unchanged caregiver assistance and mobility outcomes and worsened self-care outcomes.

**Conclusions:**

Long-term ERT in adult patients with Morquio A was associated with increased endurance and improvement in performance of ADLs.

**Trial registration:**

Trial Registration NCT01415427. Name of registry: Long-Term Efficacy and Safety Extension Study of BMN 110 in Patients With Mucopolysaccharidosis IVA (Morquio A Syndrome). Registered 8 August 2011, retrospectively registered.

**Electronic supplementary material:**

The online version of this article (doi:10.1186/s13023-017-0634-0) contains supplementary material, which is available to authorized users.

## Background

Morquio A syndrome (mucopolysaccharidosis IVA [MPS IVA]; OMIM #253000) is an autosomal recessive lysosomal storage disorder caused by deficiency in the enzyme *N*-acetylgalactosamine-6-sulfatase (GALNS; EC 3.1.6.4), resulting in impaired degradation of the glycosaminoglycans (GAGs) keratan sulfate (KS) and chondroitin-6-sulfate. Accumulation of these GAGs [1] leads to progressive development of clinical features, including skeletal and joint abnormalities, short stature, cardiorespiratory compromise, impaired vision, hearing loss, and hepatomegaly [2–4]. Estimates of the birth prevalence of this rare genetic disorder range from 1 per 76,000 to 1 per 1,179,000 [5].

The natural history of untreated Morquio A is a progressive worsening of symptoms with age [3, 6], along with declines in mobility, activities of daily living (ADLs), independence, and health related quality of life (HRQOL), as well as declining respiratory function and excessive fatigue [3, 7]. Patients with Morquio A have diminished life spans [8]. Most patients with the classical Morquio A phenotype generally die in the second to third decade of life if untreated, primarily related to cervical instability and pulmonary compromise. Approximately 25% of patients may have an attenuated, slowly progressing phenotype, with some patients reported to survive into the seventh decade of life [9].

Elosulfase alfa (Vimizim®; BioMarin Pharmaceutical Inc, Novato, CA, USA) is a recombinant human GALNS that has been recently approved as enzyme replacement therapy (ERT) for Morquio A [10]. Considering the irreversible damage caused by Morquio A, International management guidelines recommend that elosulfase alfa treatment be implemented as soon as the diagnosis of Morquio A has been confirmed [11].

The safety and efficacy of elosulfase alfa have been investigated in more than 200 patients from both randomized clinical trials and open-label extensions. In the pivotal phase III, 24-weeks, randomized, placebo-controlled study (MOR-004; NCT01275066), weekly infusions of elosulfase alfa 2.0 mg/kg significantly improved performance in the primary efficacy measure, the 6-min walk test (6MWT) distance. The long-term benefit of elosulfase alfa treatment has been demonstrated in an open-label extension trial (MOR-005; NCT01415427), in which patients from MOR-004 were enrolled for an additional 96 weeks (for a total of 120 weeks). At the end of 120 weeks, improvement in the 6MWT distance was observed in the overall population (all of whom had advanced to the 2-mg/kg/week dose by that time), as well as in the subset who received 2 mg/kg/week for the entire 120 weeks [12].

The elosulfase alfa study populations were composed largely of pediatric patients, likely due to the early mortality caused by the disease. More than 75% of the patients in MOR-004 were ≤18 years of age [13], with similar ages in MOR-005. Considering the documented importance of early treatment in MPS disorders [14–19], the benefits of initiating ERT in adults with symptoms that have already progressed is a clinically relevant issue that has not been adequately investigated. This study was a post hoc analysis of the adult population who participated in the MOR-005 extension study and specifically explored long-term clinical and patient-reported outcomes over 120 weeks in adults with Morquio A.

## Methods

### Study design

This international, multicenter, open-label trial was an extension of a randomized, double-blind, placebo-controlled, 24-weeks, phase III clinical study and was designed to evaluate the long-term efficacy and safety of elosulfase alfa treatment in patients with Morquio A (*n* = 173) [11, 13]. Details of the study design have been previously reported [12]. MOR-005 consisted of two parts. In part 1, patients assigned to 2 mg/kg/week (QW-QW cohort) and 2.0 mg/kg/every other week (QOW-QOW cohort) in MOR-004 remained on their regimen, whereas those assigned to placebo were randomized 1:1 to one of the two dosing regimens. In part 2, all patients were switched to 2.0 mg/kg/week. (Study design presented in Additional file 1: Figure S1.)

### Patient selection

Selection criteria for MOR-005 have been previously reported [12]. This report was a post hoc subanalysis of the adult patients (≥18 years of age) who participated in MOR-005.

### Efficacy evaluation

The primary efficacy measure was distance (m) walked in the 6MWT, which evaluates functional exercise capacity and endurance. Secondary endpoints included the 3-min stair climb test (3MSCT), also a measure of endurance, and urinary KS (uKS) levels normalized to urine creatinine. Respiratory function was assessed by forced vital capacity, forced expiratory volume in 1 s, and maximal voluntary ventilation tests in accordance with the American Thoracic Society guidelines [20]. The Health Assessment Questionnaire adapted for patients with MPS (MPS HAQ) [3] assessed ADLs and extent of disability in the domains of self-care, mobility, and amount of caregiver assistance required. Higher numbers on the HAQ domain scores indicate greater impairment. The individual items on the self-care and mobility domains were rated from 0 (not difficult at all) to 11 (unable to do) and averaged to provide a domain score also ranging from 0 to 11. The items on the caregiver assistance domain were rated from 1 to 4 and were summed together to provide a score ranging from 13 to 52.

The placebo group from MOR-004 was transitioned to QW or QOW dosing after the 24-weeks double-blind phase, and therefore no placebo group was included in MOR-005. However, to provide context for interpretation of the results, data from untreated patients were obtained from the Morquio A Clinical Assessment Program (MorCAP) natural history study (MOR-001; NCT00787995). MorCAP was an international, multicenter, cross-sectional study, subsequently amended as a longitudinal study [3, 7]. The comparison group for this report consisted of individuals who were ≥18 years of age at baseline, had a mean 6MWT distance ≥30 m to ≤325 m, and had 2-years follow-up data.

### Safety evaluation

A detailed description of safety assessments performed during MOR-005 has been previously reported and included adverse events (AEs), vital signs, concomitant medications, infusion-associated reactions, clinical laboratory results, physical exam results, and immunogenicity testing results [12].

### Statistical methods

In addition to the intent-to-treat (ITT) population in MOR-005, a modified per-protocol (MPP) population analysis was performed to address the impact of orthopedic surgical procedures and noncompliance with study protocol. Because orthopedic surgical procedures and the subsequent recovery period can affect mobility and endurance tests, both positively and negatively, patients who underwent orthopedic operations during the study were excluded from the MPP analysis. Patients were also excluded from the MPP population for noncompliance, defined as missing ≥20% of scheduled elosulfase alfa infusions during MOR-005. Descriptive statistics of the efficacy endpoints are provided for the ITT and MPP populations, both pooled and by treatment cohort (Additional files 2 and 3: Tables S1 and S2). Comparison of least squares (LS) mean efficacy assessments between week 120 and baseline used a repeated-measures analysis of covariance model. For the 6MWT, the model included baseline 6MWT category (< or ≥200 m), treatment, time point, and treatment and time point interaction. For 3MSCT and uKS level, the models also included baseline measurement for 3MSCT and uKS level, respectively. For all other assessments, baseline measurement for the respective assessment replaced baseline 6MWT category. The same models were used to compare year 2 and baseline for the respective assessments in the MorCAP population. The safety analysis included patients who received any dose of study drug and had any posttreatment safety assessments (ITT population). The incidence of AEs was summarized by severity and relation to study drug.

## Results

### Patient characteristics

The patient characteristics for the overall population in MOR-005 have been published previously [12]. Of the 39 patients ≥18 years of age in the MOR-004 ITT population, one discontinued early in the study (day 16) for personal reasons. Thirty-eight patients completed MOR-004 and 37 continued into MOR-005; 32 of these patients were included in the MPP population for both studies. During MOR-005, one patient discontinued due to safety and compliance issues (ITT population only) and four discontinued due to study termination by the sponsor (both ITT and MPP populations).

To serve as a comparator, patients were selected from the MorCAP study of the natural history of untreated Morquio A syndrome [7]. Of the 353 patients in MorCAP, 10 were ≥18 years of age, with 2-years follow-up data, and met the inclusion criteria for MOR-004/005. The number of patients with data available at each assessment ranged from 4 to 10. No patients from this MorCAP subpopulation underwent orthopedic operations during the relevant time period. Therefore, data from the same group of patients were used for comparison with the MOR-005 ITT and MPP populations.

Due to the observational nature of the natural history study, the exact interval of follow-up visits varied between 638 and 939 days (91–134 weeks) for the year 2 assessment. The mean (SD) actual visit time was 773 (102) days, or approximately 110 weeks. Table 1 shows the demographics and baseline characteristics for the adults enrolled in MOR-005, as well as the corresponding subpopulation from the MorCAP study. Adult patients in MOR-004/005 had mean baseline height, endurance, and pulmonary function measures that were higher than those in MorCAP, whereas the MorCAP patients were younger than the MOR-004/005 patients; baseline MPS-HAQ scores were similar between groups. Although their baseline mean height (114.1 cm, ITT) is characteristic of a severely affected group, the adult patients in MOR-004/005 were still taller than the MOR-004 overall population (mean baseline range, 101–105 cm), indicating that the disease in these adults was slightly attenuated, as is consistent with their prolonged survival [13].

**Table 1:** Patient demographics and baseline characteristics of patients ≥18 years of age in MOR-004/005 and MorCAP

	ITT (*n* = 37)	MPP (*n* = 32)	MorCAP (*n* = 10)^a^
Demographics			
Age, mean (SD), yrs	30.9 (9.7)	30.3 (8.5)	26.9 (7.8)
Male, *n* (%)	13 (35.1)	12 (37.5)	1 (10.0)
Ethnicity, n (%)			
White	30 (81.1)	26 (81.3)	8 (80.0)
Nonwhite	7 (18.9)	6 (18.8)	2 (20.0)
Height, mean (SD), cm	114.1 (20.4)	114.6 (20.8)	106.4 (13.1)
Baseline characteristics, mean (SD)			
6MWT distance, m	176.9 (67.3)	175.3 (70.7)	148.6 (109.1)
3MSCT, stairs/min	21.3 (13.1)	22.6 (13.2)	16.6 (15.3)
uKS, μg/mg	9.5 (6.2)	9.6 (6.5)	7.2 (5.0)
FVC, L	1.5 (1.1)	1.5 (1.1)	0.9 (0.3)
FEV_1_, L	1.2 (0.8)	1.3 (0.9)	0.7 (0.3)
MVV, L/min	43.2 (31.7)	44.9 (33.9)	29.8 (15.2)
HAQ—self-care	2.7 (2.2)	2.0 (1.9)	2.4 (1.9)
HAQ—caregiver assistance	23.0 (10.0)	23.7 (10.5)	25.8 (8.0)
HAQ—mobility	5.4 (2.7)	5.5 (2.5)	5.4 (2.4)

*3MSCT* 3-min stair climb test, *6MWT* 6-min walk test, *FEV*_*1*_ forced expiratory volume in 1 s, *FVC* forced vital capacity, *HAQ* Health Assessment Questionnaire, *ITT* intent to treat, *MPP* modified per-protocol, *MVV* maximal voluntary ventilation, *uKS* urinary keratan sulfate

^a^ *n* = 9 for 6MWT, 3MSCT, FVC, and FEV_1_; *n* = 7 for MVV; *n* = 4 for uKS. Includes only patients with data at year 2

### Primary efficacy measure

**Fig. 1:**
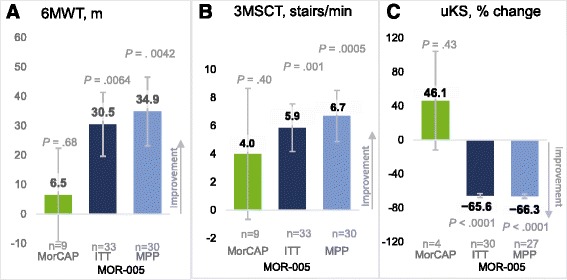
LS mean change from baseline for endurance and uKS level. LS mean change from baseline to week 120 (MOR-005) or year 2 (MorCAP) in (**a**) 6MWT, (**b**) 3MSCT, and (**c**) uKS level. Error bars represent standard error. *P* values were calculated based on comparison of LS mean assessments at week 120 or year 2 vs baseline. 3MSCT, 3-min stair climb test; 6MWT, 6-min walk test; ITT, intent to treat; LS, least squares; MPP, modified per-protocol; uKS, urinary keratan sulfate

For adult patients in MOR-005, the LS mean (SE) increase from MOR-004 baseline to week 120 in the 6MWT was 30.5 (10.8) m for the ITT population (*P* = .0064) and 34.9 (11.7) m for the MPP group (*P* = .0042) (Fig. 1a; Additional file 4: Table S3). In comparison, the MorCAP patients (*n* = 10) did not show improvement over 2 years, with an LS mean change of 6.5 m, well within the SE of 15.9 m. Of the ITT population, a subset of 10 patients (34.4%) received the approved 2-mg/kg QW dose in both MOR-004 and MOR-005 for a total of 120 weeks. The mean (SE) change in the 6MWT distance at week 120 for this cohort receiving the more efficacious dose throughout the study was 54.4 (9.9) m, with a similar change of 57.6 (10.5) m in the MPP (*n* = 9) population, further supporting the selection of 2 mg/kg QW as the appropriate dosing regimen (Additional file 3: Table S2).

### Secondary outcome measures

**Fig. 2:**
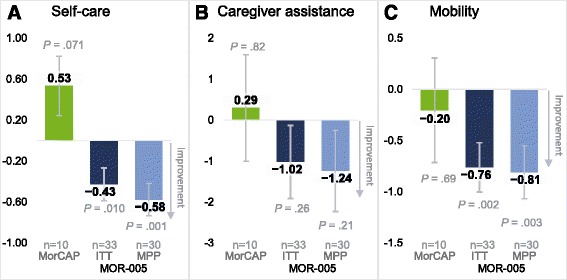
LS mean change from baseline for MPS HAQ domain scores. LS mean change from baseline to week 120 (MOR-005) or year 2 (MorCAP) in the MPS HAQ domains of (**a**) self-care, (**b**) caregiver assistance, and (**c**) mobility. Error bars represent standard error. *P* values were calculated based on comparison of LS mean assessments at week 120 or year 2 vs baseline. ITT, intent to treat; LS, least squares; MPP, modified per-protocol; MPS HAQ, Mucopolysaccharidosis Health Assessment Questionnaire

**Fig. 3:**
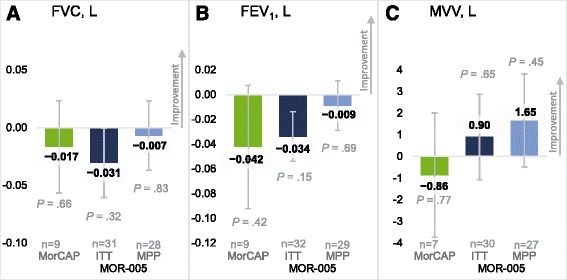
LS mean change from baseline for respiratory tests. LS mean change from baseline to week 120 (MOR-005) or year 2 (MorCAP) in (**a**) FVC, (**b**) FEV_1_, and (**c**) MVV. Error bars represent standard error. *P* values were calculated based on comparison of LS mean assessments at week 120 or year 2 vs baseline. FEV_1_, forced expiratory volume in 1 s; FVC, forced vital capacity; ITT, intent to treat; LS, least squares; MPP, modified per-protocol; MVV, maximal voluntary ventilation

The adult patients in MOR-005 demonstrated improvements from baseline to week 120 as measured by the 3MSCT, uKS level, and the MPS HAQ domains of self-care, caregiver assistance, and mobility (Figs. 1 and 2; Additional file 2: Table S1). The LS mean (SE) change in 3MSCT at week 120 was 5.9 (1.7) stairs/min in the ITT population (*n* = 33; *P* = .0010) and 6.7 (1.8) stairs/min in the MPP population (*n* = 30; *P* = .0005) (Fig. 1b; Additional file 4: Table S3). In MorCAP (*n* = 9), the 3MSCT showed no meaningful improvement through year 2, with an LS mean (SE) change of 4.0 (4.7) stairs/min. The uKS level decreased in the MOR-005 study (*n* = 30, ITT), with an LS mean (SE) percentage change of −65.6% (2.4%), in contrast to an increase observed in MorCAP (*n* = 4) of 46.1% (58.1%) (Fig. 1c; Additional file 4: Table S3). However, because uKS level decreases with age, this increase is likely a result of minor fluctuations in absolute uKS level relative to the lower baseline uKS level (Additional file 3: Table S2). The MPS HAQ domain score of self-care improved at week 120, with a LS mean change (SE) of −0.43 (0.2) in the ITT population (*n* = 33; *P* = .010) and −0.58 (0.2) in the MPP population (*n* = 30; *P* = .001) (Fig. 2a; Additional file 4: Table S3). In the MorCAP sample (*n* = 10), the self-care domain score worsened, with LS mean change (SE) of 0.53 (0.3). On the caregiver assistance domain score, the improvement was −1.02 (0.9) in the MOR-005 ITT population (*n* = 33) (Additional file 4: Table S3); on the mobility domain score, the improvement was −0.76 (0.24) (Additional file 4: Table S3). Similar results were observed in the MPP analysis (Fig. 2b and c). Results of the MorCAP patients (*n* = 10) were too variable to be conclusive on these two measures, with LS mean (SE) changes of 0.29 (1.3) and −0.20 (0.5) on the caregiver and mobility domain scores, respectively. No significant changes were detected on respiratory tests for the adult patients of either study over this time period (Fig. 3; Supplemental Table S3).

### Safety

AEs and serious AEs reported for adult patients (ITT population) during MOR-005 were consistent with those of the overall study population (Additional file 5: Table S4). All adult patients experienced an AE, with the majority being of mild to moderate severity; 73% of AEs in the adult population were considered related to study drug. Although most adult patients (97.3%) experienced one or more infusion-associated reaction, only 0.4% of their infusions were interrupted or discontinued because of an AE requiring medical intervention.

### Immunogenicity

Immunogenicity findings for the adult population did not differ from those previously reported for the overall study population [12]. Briefly, all patients treated with elosulfase alfa developed both antidrug antibodies and neutralizing antibodies (antibodies capable of interfering with CI-M6PR binding in vitro; nAb). No association was detected between higher total antidrug antibody titer or nAb positivity and reduced efficacy measurements (6MWT, 3MSCT, and uKS level) or the incidence or severity of hypersensitivity AEs. Drug-specific immunoglobulin E was detected in 2 of 37 patients (5.4%); however, positivity was not associated with the occurrence of anaphylaxis or other hypersensitivity AEs.

## Discussion

This subanalysis of a long-term open-label study showed that treating adults with elosulfase alfa over 120 weeks led to improvements in 6MWT, 3MSCT, and ADL performance, as well as reductions in uKS level. Although no improvement was seen in pulmonary function, this has been reported to decline in patients >14 years of age [6] suggesting that treatment may still have positively impacted disease progression. The lack of decline in the pulmonary function of MorCAP subjects was in conflict with the previously reported findings [6] and may be related to the relatively small sample size.

HRQOL in adults with Morquio A has been shown to be most negatively affected in the domains of mobility, self-care, and usual activities (eg, work, study, housework, family, or leisure activities) and drops dramatically when patients become wheelchair bound [7]. In this report, treated adults improved on the MPS HAQ domain scores of both mobility and self-care, whereas MorCAP scores showed a trend toward decline in the self-care domain (*P* = .07), with no change in mobility over a similar period of time.

As has been previously noted [12], the interpretation of results from the MOR-005 long-term study is complicated by three main factors: the staggered transition to QW dosing regimen, occurrence of orthopedic surgical procedures during the study, and the absence of a placebo group.

Patients were transitioned from 2.0 mg/kg QOW dosing to QW dosing as soon as it was identified as the optimal regimen, but the resulting staggered dosing transition contributed to the complexity of the study and precluded a meaningful analysis of QOW dosing efficacy. However, 9 adult patients from the MPP analysis were treated at the optimal dose of 2.0 mg/kg QW throughout the 120 weeks, and these patients improved on 6MWT distance by 57.6 m (compared with the 34.9-m improvement for all adults combined), a 31% increase from the mean baseline of 186.1 m.

The exclusion of patients who underwent orthopedic operations from the MPP analysis population had the potential to bias results by removing more severely affected patients. However, a comparison between the ITT and MPP populations at baseline showed that the groups were similar in all measures, including height, uKS level, and endurance (Table 1), suggesting that there was no difference in disease severity between the ITT and MPP groups.

In the absence of a placebo group for the extension study, data were placed in the context of the progressive nature of untreated Morquio A through comparison with data from the MorCAP natural history study, with similar inclusion and exclusion criteria applied retrospectively. However, the differences in baseline demographics between the studies and the small sample size available from the natural history study limit the interpretation of these comparisons.

Although the benefits of early ERT treatment for patients with Morquio A have been well established [11–13], to our knowledge, this is the first report of long-term outcomes of initiating ERT in adult patients with established disease. As expected, disease symptoms at baseline appeared to be more severe in the adult subgroup than in the overall population, with a baseline 6MWT distance of 176.9 m (Table 1) vs 209.5 m for the overall MOR-004/MOR-005 ITT population [12]. However, improvements in multiple efficacy and HRQOL measures were observed over the 120-weeks study of elosulfase alfa, suggesting that adults with established disease derived benefits from ERT initiation and treatment.

## Conclusions

The patients with Morquio A in this report were adults and, as such, had already experienced ≥18 years of multisystemic GAG accumulation and tissue damage. In this population, ERT with elosulfase alfa led to improvements in endurance, mobility, and self-care that were sustained over 120 weeks. Analysis of similar patients from the MorCAP natural history study suggested that untreated adult patients do not typically experience improvements in these outcomes over a similar time interval. The long-term improvements observed in the MPS HAQ domain scores of self-care and mobility in particular indicate that adult patients may experience improvements in HRQOL with elosulfase alfa treatment. Furthermore, no new or unexpected safety signals were identified in this patient group. Collectively, these results support initiating ERT in adult patients with Morquio A and are consistent with international guidelines [11], which recommend implementing elosulfase alfa ERT for all patients with a confirmed diagnosis of Morquio A to replace deficient GALNS.

## Additional files

Additional file 1: Figure S1. Study design. After completion of MOR-004, patients initially randomized to 2.0 mg/kg/week (QW-QW cohort) or 2.0 mg/kg every other week (QOW-QOW cohort) remained on their assigned dosing regimen. Patients taking placebo were randomized to one of the two dosing regimens (PBO-QOW or PBO-QW cohort, respectively). After a review of efficacy and safety results from MOR-004 established the recommended dose for part 2, all patients were switched to 2.0 mg/kg QW. Specific week of transition ranged from week 36–96, depending on enrollment timing. (PDF 866 kb)

Additional file 2: Table S1. Descriptive statistics on change from baseline to year 2 (MorCAP) or week 120 (MOR-005). (PDF 74 kb)

Additional file 3: Table S2. Descriptive statistics on change from baseline to year 2 (MOR-001) or week 120 (MOR-005) by dosing cohort. (PDF 89 kb)

Additional file 4: Table S3. LS mean change from baseline to year 2 (MorCAP) or week 120 (MOR-005) based on a repeated-measures ANCOVA model^a^. (PDF 84 kb)

Additional file 5: Table S4. Summary of adverse events. (PDF 64 kb)

## Abbreviations

3MSCT: 3-minutes stair climb test
6MWT: 6-minutes walk test
ADLs: Activities of daily living
AEs: Adverse events
ERT: Enzyme replacement therapy
GAGs: Glycosaminoglycans
GALNS: Acetylgalactosamine-6-sulfatase
HRQOL: Health related quality of life
ITT: Intent-to-treat
KS: Keratan sulfate
MPP: Modified per protocol
MPS HAQ: MPS health assessment questionnaire
MPS: Mucopolysaccharidosis IVA
uKS: urinary KS
