# Supplementary material for: Clinical outcomes in a subpopulation of adults with Morquio A syndrome: results from a long-term extension study of elosulfase alfa
Source: Orphanet J Rare Dis. 2017 May 23;12:98. doi: 10.1186/s13023-017-0634-0 (PMC5442692; doi:10.1186/s13023-017-0634-0)
Supplement: Supplementary file 1 — Study design. After completion of MOR-004, patients initially randomized to 2.0 mg/kg/week (QW-QW cohort) or 2.0 mg/kg every other week (QOW-QOW cohort) remained on their assigned dosing regimen. Patients taking placebo were randomized to one of the two dosing regimens (PBO-QOW or PBO-QW cohort, respectively). After a review of efficacy and safety results from MOR-004 established the recommended dose for part 2, all patients were switched to 2.0 mg/kg QW. Specific week of transition ranged from week 36–96, depending on enrollment timing. (PDF 866 kb) [file 13023_2017_634_MOESM1_ESM.pdf]

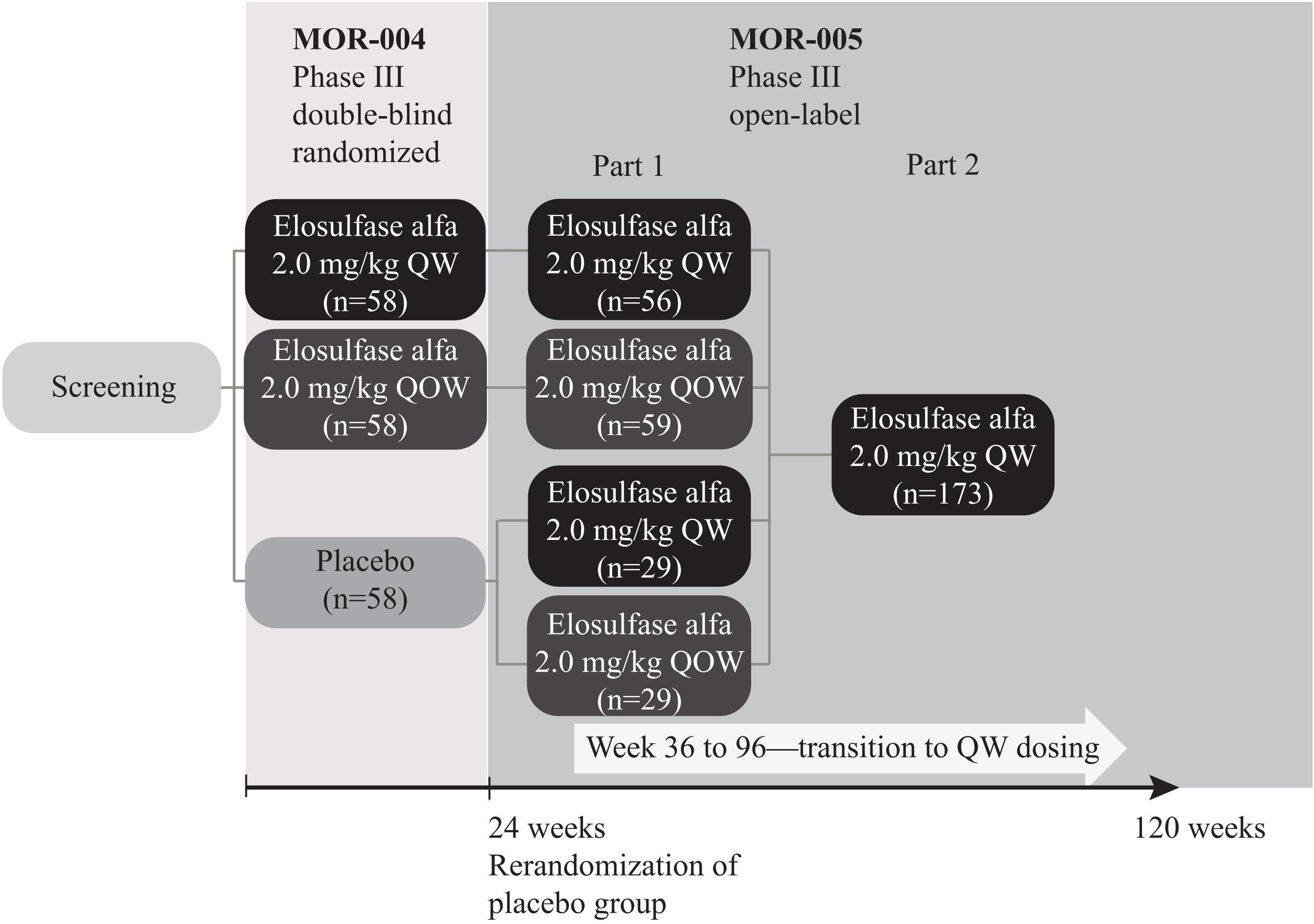

Fig. S1 Study design. After completion of MOR-004, patients initially randomized to 2.0 mg/kg/week (QW-QW cohort) or 2.0 mg/kg every other week (QOW-QOW cohort) remained on their assigned dosing regimen. Patients taking placebo were randomized to one of the two dosing regimens (PBO-QOW or PBO-QW cohort, respectively). After a review of efficacy and safety results from MOR-004 established the recommended dose for part 2, all patients were switched to 2.0 mg/kg QW. Specific week of transition ranged from week 36 to 96, depending on enrollment timing.
