# Supplementary material for: Clinical outcomes in a subpopulation of adults with Morquio A syndrome: results from a long-term extension study of elosulfase alfa
Source: Orphanet J Rare Dis. 2017 May 23;12:98. doi: 10.1186/s13023-017-0634-0 (PMC5442692; doi:10.1186/s13023-017-0634-0)
Supplement: Supplementary file 2 — Descriptive statistics on change from baseline to year 2 (MorCAP) or week 120 (MOR-005). (PDF 74 kb) [file 13023_2017_634_MOESM2_ESM.pdf]

Table S1. Descriptive statistics on change from baseline to year 2 (MorCAP) or week 120 (MOR-005).

Measure	Study	Analysis	N	Mean change	SE
**6MWT, m**	MorCAP	ITT	9	4.3	10.5
	MOR-005	ITT	33	29.6	13.8
		MPP	30	34.1	14.7
**3MSCT, stairs/min**	MorCAP	ITT	9	3.4	5.2
	MOR-005	ITT	33	5.8	2.0
		MPP	30	6.6	2.1
**uKS , µg/mg[a]**	MorCAP	ITT	4	3.0	1.9
	MOR-005	ITT	30	−6.4	0.9
		MPP	27	−6.6	0.9
**uKS , % change[a]**	MorCAP	ITT	4	72.3	37.3
	MOR-005	ITT	30	−65.4	2.2
		MPP	27	−66.0	2.4
**FVC, L**	MorCAP	ITT	9	−0.002	0.05
	MOR-005	ITT	31	0.0	0.04
		MPP	28	0.0	0.04
**FVC % change**	MorCAP	ITT	9	−2.0	3.3
	MOR-005	ITT	31	−2.6	2.7
		MPP	28	−0.7	2.8
**FEV_1, L**	MorCAP	ITT	9	−0.039	0.047
	MOR-005	ITT	32	0.0	0.04
		MPP	29	0.0	0.02
**FEV_1 % change**	MorCAP	ITT	9	−4.2	3.8
	MOR-005	ITT	32	−1.9	2.9
		MPP	29	0.5	2.8
**MVV, L/min**	MorCAP	ITT	7	−1.40	3.8
	MOR-005	ITT	30	0.9	2.2
		MPP	27	1.7	2.3
**MVV % change**	MorCAP	ITT	7	−1.9	7.7
	MOR-005	ITT	30	1.7	4.5
		MPP	27	3.7	4.6
**Self-care**	MorCAP	ITT	10	0.5	0.5
	MOR-005	ITT	33	−0.4	0.2
		MPP	30	−0.6	0.2
**Caregiver assistance change**	MorCAP	ITT	10	0.4	2.3
	MOR-005	ITT	33	−1.2	1.1
		MPP	30	−1.3	1.2
**Mobility change**	MorCAP	ITT	10	−0.3	1.0
	MOR-005	ITT	33	−0.8	0.3
		MPP	30	−0.8	0.3

3MSCT, 3-minute stair climb test; 6MWT, 6-minute walk test; FEV_1, forced expiratory volume in 1 second; FVC, forced vital capacity; HAQ, Health Assessment Questionnaire; ITT, intent to treat; MPP, modified per-protocol; MVV, maximal voluntary ventilation; uKS, urinary keratan sulfate.

[a]Normalized uKS is calculated as uKS divided by urine creatinine.
