# Supplementary material for: Clinical outcomes in a subpopulation of adults with Morquio A syndrome: results from a long-term extension study of elosulfase alfa
Source: Orphanet J Rare Dis. 2017 May 23;12:98. doi: 10.1186/s13023-017-0634-0 (PMC5442692; doi:10.1186/s13023-017-0634-0)
Supplement: Supplementary file 3 — Descriptive statistics on change from baseline to year 2 (MOR-001) or week 120 (MOR-005) by dosing cohort. (PDF 89 kb) [file 13023_2017_634_MOESM3_ESM.pdf]

Table S2. Descriptive statistics on change from baseline to year 2 (MOR-001) or week 120 (MOR-005) by dosing cohort.

				MOR-005			
**Measure**	**Analysis**	**Parameter**	**MOR-001**	**PBO-QOW**	**PBO-QW**	**QOW-QOW**	**QW-QW**
**6MWT change, m**	ITT	N	9	7	6	10	10
		Mean (SE)	4.3 (10.5)	34.2 (23.9)	51.3 (20.8)	−11.4 (37.8)	54.4 (9.9)
	MPP	N		5	6	10	9
		Mean (SE)		62.1 (20.1)	51.3 (20.8)	−11.4 (37.8)	57.6 (10.5)
**3MSCT, stairs/min**	ITT	N	9	7	6	10	10
		Mean (SE)	3.4 (5.2)	2.1 (1.8)	8.2 (6.2)	7.4 (4)	5.3 (3.6)
	MPP	N		5	6	10	9
		Mean (SE)		4.2 (1.6)	8.2 (6.2)	7.4 (4)	6.2 (3.9)
**uKS, µg/mg**[a]	ITT	N	4	7	4	9	10
		Mean (SE)	3.0 (1.9)	−3.7 (0.8)	−8.4 (2.7)	−4.6 (0.9)	−9.2 (1.9)
	MPP	N		5	4	9	9
		Mean (SE)		−3.8 (1.1)	−8.4 (2.7)	−4.6 (0.9)	−9.2 (2.1)
**uKS, % change**	ITT	N	4	7	4	9	10
		Mean (SE)	72.3 (37.3)	−64.3 (4.6)	−64.4 (5)	−62.6 (4.1)	−69 (4.1)
	MPP	N		5	4	9	9
		Mean (SE)		−66.9 (6.1)	−64.4 (5.0)	−62.6 (4.1)	−69.6 (4.6)
**FVC, L**	ITT	N	9	8	6	10	7
		Mean (SE)	−0.002 (0.05)	−0.106 (0.07)	−0.042 (0.05)	0.024 (0.06)	−0.024 (0.11)
	MPP	N		6	6	10	6
		Mean (SE)		−0.032 (0.07)	−0.042 (0.05)	0.024 (0.06)	−0.013 (0.13)
**FVC, % change**	ITT	N	9	8	6	10	7
		Mean (SE)	−2.0 (3.3)	−4.4 (4.5)	1.1 (6.0)	1.2 (4.3)	−9.1 (7.6)
	MPP	N		6	6	10	6
		Mean (SE)		1.2 (3.4)	1.1 (6.0)	1.2 (4.3)	−7.4 (8.8)
**FEV_1, L**	ITT	N	9	8	6	10	8
		Mean (SE)	−0.039 (0.047)	−0.119 (0.072)	0.063 (0.024)	−0.044 (0.022)	−0.015 (0.075)
	MPP	N		6	6	10	7
		Mean (SE)		−0.032 (0.058)	0.063 (0.024)	−0.044 (0.022)	−0.007 (0.086)
**FEV_1, % change**	ITT	N	9	8	6	10	8
		Mean (SE)	−4.2 (3.8)	−6.7 (3.2)	9.0 (3.6)	−3.6 (3.4)	3.3 (9.8)
	MPP	N		6	6	10	7
		Mean (SE)		0.9 (3.2)	9.0 (3.6)	−3.6 (3.4)	−1.3 (9.8)
**MVV, L**	ITT	N	7	8	5	10	7
		Mean (SE)	−1.40 (3.8)	−5.43 (5.6)	6.89 (7.4)	2.77 (2.9)	1.2 (1.4)
	MPP	N		6	5	10	6
		Mean (SE)		−3.81 (7.2)	6.89 (7.4)	2.77 (2.9)	1.08 (1.6)
**MVV, % change**	ITT	N	7	8	5	10	7
		Mean (SE)	−1.9 (7.7)	−1.1 (10.7)	3.3 (14.9)	1.6 (6)	4.1 (8.9)
	MPP	N		6	5	10	6
		Mean (SE)		7.9 (10.9)	3.3 (14.9)	1.6 (6.0)	3.3 (10.5)
**HAQ—self-care domain change**	ITT	N	10	8	5	10	10
		Mean (SE)	0.5 (0.5)	−0.1 (0.5)	−0.8 (0.6)	−0.6 (0.3)	−0.4 (0.3)
	MPP	N		6	5	10	9
		Mean (SE)		−0.5 (0.4)	−0.8 (0.6)	−0.6 (0.3)	−0.5 (0.4)
**HAQ—caregiver assistance**	ITT	N	10	8	5	10	10
		Mean (SE)	0.4 (2.3)	−2.4 (2.5)	−0.2 (2.0)	−1.6 (2.3)	−0.2 (1.7)
	MPP	N		6	5	10	9
		Mean (SE)		−2.5 (3.4)	−0.2 (2)	−1.6 (2.3)	−0.9 (1.7)
**HAQ—mobility change**	ITT	N	10	8	5	10	10
		Mean (SE)	−0.3 (1.0)	−0.8 (0.4)	−1.6 (1.4)	−0.7 (0.4)	−0.4 (0.5)
	MPP	N		6	5	10	9
		Mean (SE)		−0.8 (0.4)	−1.6 (1.4)	−0.7 (0.4)	−0.5 (0.6)

3MSCT, 3-minute stair climb test; 6MWT, 6-minute walk test; FEV_1, forced expiratory volume in 1 second; FVC, forced vital capacity; HAQ, Health Assessment Questionnaire; ITT, intent to treat; MPP, modified per-protocol; MVV, maximal voluntary ventilation; uKS, urinary keratan sulfate

[a]Normalized uKS is calculated as urine keratan sulfate divided by urine creatinine
