# Supplementary material for: Clinical outcomes in a subpopulation of adults with Morquio A syndrome: results from a long-term extension study of elosulfase alfa
Source: Orphanet J Rare Dis. 2017 May 23;12:98. doi: 10.1186/s13023-017-0634-0 (PMC5442692; doi:10.1186/s13023-017-0634-0)
Supplement: Supplementary file 4 — LS mean change from baseline to year 2 (MorCAP) or week 120 (MOR-005) based on a repeated-measures ANCOVA modela. (PDF 84 kb) [file 13023_2017_634_MOESM4_ESM.pdf]

Table S3. LS mean change from baseline to year 2 (MorCAP) or week 120 (MOR-005) based on a repeated-measures ANCOVA model[a].

Measure	Study	Analysis	N	LS mean change	SE	*P* value[b]
**6MWT, m**	MorCAP	ITT	9	6.5	15.9	.68
	MOR-005	ITT	33	30.5	10.8	.0064
		MPP	30	34.9	11.7	.0042
**3MSCT, stairs/min**	MorCAP	ITT	9	4.0	4.7	.40
	MOR-005	ITT	33	5.9	1.7	.001
		MPP	30	6.7	1.8	.0005
**uKS % change**	MorCAP	ITT	4	46.1	58.1	.43
	MOR-005	ITT	30	−65.6	2.4	<.0001
		MPP	27	−66.3	2.7	<.0001
**FVC, L**	MorCAP	ITT	9	−0.017	0.04	.66
	MOR-005	ITT	31	−0.031	0.03	.32
		MPP	28	−0.007	0.03	.83
**FEV_1, L**	MorCAP	ITT	9	−0.042	0.05	.42
	MOR-005	ITT	32	−0.034	0.02	.15
		MPP	29	−0.009	0.02	.69
**MVV change, L/min**	MorCAP	ITT	7	−0.86	2.9	.77
	MOR-005	ITT	30	0.90	2.0	.65
		MPP	27	1.65	2.2	.45
**Caregiver assistance change**	MorCAP	ITT	10	0.29	1.3	.82
	MOR-005	ITT	33	−1.02	0.9	.26
		MPP	30	−1.24	1.0	.21
**Mobility change**	MorCAP	ITT	10	−0.20	0.5	.69
	MOR-005	ITT	33	−0.76	0.2	.002
		MPP	30	−0.81	0.3	.003
**Self-care**	MorCAP	ITT	10	0.53	0.3	.071
	MOR-005	ITT	33	−0.43	0.2	.010
		MPP	30	−0.58	0.2	.001

3MSCT, 3-minute stair climb test; 6MWT, 6-minute walk test; ANCOVA, analysis of covariance; FEV_1, forced expiratory volume in 1 second; FVC, forced vital capacity; ITT, intent to treat; LS, least squares; MPP, modified per-protocol; MVV, maximal voluntary ventilation; uKS, urinary keratan sulfate.

[a]For 6MWT, the model included baseline 6MWT category (< or ≥200 m), treatment, time point, and treatment and time point interaction. For all other assessments, baseline measurement replaced baseline 6MWT category, except for 3MSCT and uKS, which included both terms.

[b]*P* values were calculated for LS mean assessments at year 2 or week 120 vs baseline.
