# Supplementary material for: Clinical outcomes in a subpopulation of adults with Morquio A syndrome: results from a long-term extension study of elosulfase alfa
Source: Orphanet J Rare Dis. 2017 May 23;12:98. doi: 10.1186/s13023-017-0634-0 (PMC5442692; doi:10.1186/s13023-017-0634-0)
Supplement: Supplementary file 5 — Summary of adverse events. (PDF 64 kb) [file 13023_2017_634_MOESM5_ESM.pdf]

Table S4. Summary of adverse events.

Adverse event	ITT (N=173)	≥18 years ITT (N=37)	MPP (N=124)	≥18 years MPP (N=32)
**Any AE**, n (%)	173 (100.0)	37 (100.0)	124 (100.0)	32 (100.0)
**Grade 1**	33 (19.1)	6 (16.2)	29 (23.4)	6 (18.8)
**Grade 2**	89 (51.4)	23 (62.2)	66 (53.2)	21 (65.6)
**Grade 3**	44 (25.4)	7 (18.9)	27 (21.8)	4 (12.5)
**Grade 4**	6 (3.5)	1 (2.7)	2 (1.6)	1 (3.1)
**Grade 5**	1 (0.6)	0	0	0
**Number of AEs per patient, mean/median**	35.3/28.0	40.6/28.0	36.0/28.5	39.7/28.5
**Any study drug–related AE**, n (%)[a]	128 (74.0)	27 (73.0)	89 (71.8)	23 (71.9)
**Grade 1**	64 (37.0)	13 (35.1)	46 (37.1)	11 (34.4)
**Grade 2**	60 (34.7)	14 (37.8)	41 (33.1)	12 (37.5)
**Grade 3**	3 (1.7)	0 (0.0)	2 (1.6)	0 (0.0)
**Grade 4**	1 (0.6)	0	0	0
**Any SAE**, n (%)	77 (44.5)	14 (37.8)	35 (28.2)	9 (28.1)
**Grade 1**	11 (6.4)	1 (2.7)	5 (4.0)	1 (3.1)
**Grade 2**	25 (14.5)	6 (16.2)	11 (8.9)	4 (12.5)
**Grade 3**	34 (19.7)	6 (16.2)	17 (13.7)	3 (9.4)
**Grade 4**	6 (3.5)	1 (2.7)	2 (1.6)	1 (3.1)
**Grade 5**	1 (0.6)	0 (0.0)	2 (1.6)	0
**Number of SAEs per patient, mean/median**	0.7/0.0	0.5/0.0	0.4/0.0	0.4/0.0
**Any study drug–related SAE**, n (%)[a]	2 (1.2)	0	1 (0.8)	0
**Grade 2**	1 (0.6)	0	1 (0.8)	0
**Grade 4**	1 (0.6)	0	0	0
**Any AE leading to study discontinuation**, n (%)	3 (1.7)	0	0	0
**Grade 3**	1 (0.6)	0	0	0
**Grade 4**	1 (0.6)	0	0	0
**Grade 5**	1 (0.6)	0	0	0
**Any AE leading to permanent study drug discontinuation**, n (%)	4 (2.3)	1 (2.7)	1 (0.8)	1 (3.1)
**Grade 3**	2 (1.2)	1 (2.7)	1 (0.8)	1 (3.1)
**Grade 4**	1 (0.6)	0	0	0
**Grade 5**	1 (0.6)	0	0	0
**Death**	1 (0.6)	0	0	0

SAE, serious adverse event.
